# Genome-wide SNP analysis using 2b-RAD sequencing identifies the candidate genes putatively associated with resistance to ivermectin in *Haemonchus contortus*

**DOI:** 10.1186/s13071-016-1959-6

**Published:** 2017-01-17

**Authors:** Xiaoping Luo, Xiaona Shi, Chunxiu Yuan, Min Ai, Cheng Ge, Min Hu, Xingang Feng, Xiaoye Yang

**Affiliations:** 1 Shanghai Veterinary Research Institute, Chinese Academy of Agricultural Sciences, Key Laboratory of Animal Parasitology, Ministry of Agriculture of China, Shanghai, 200241 People’s Republic of China; 2 College of Veterinary Medicine, Inner Mongolia Agricultural University, Hohhot, 010010 Inner Mongolia Nationality Autonomous People’s Republic of China; 3 College of Life and Environmental Sciences, Shanghai Normal University, Shanghai, 250014 People’s Republic of China; 4 State Key Laboratory of Agricultural Microbiology, Key Laboratory of Development of Veterinary Diagnostic Products, Ministry of Agriculture, College of Veterinary Medicine, Huazhong Agricultural University, 1 Shizishan Street, Wuhan, 430070 Hubei Province People’s Republic of China; 5 Jiangsu Co-innovation Center for Prevention and Control of Important Animal Infectious Diseases and Zoonoses, Yangzhou, 225009 Jiangsu Province People’s Republic of China

**Keywords:** Candidate ivermectin resistance-associate genes, *Haemonchus contortus*, 2b-RAD sequencing, Genome-wide SNP analysis

## Abstract

**Background:**

The excessive and uncontrolled use of anthelmintics, e.g. ivermectin (IVM) for the treatment of livestock parasites has led to widespread resistance in gastrointestinal nematodes, such as *Haemonchus contortus*. There is an urgent need for better management of drug-use in nematode control and development of novel anthelmintics. Discovery and identification of anthelmintic resistance-associate molecules/markers can provide a basis for rational anthelmintics-use and development of novel drugs. Recent studies have shown that ivermectin resistance in *H. contortus* is likely to be multi-genic in nature except for several genes coding for IVM target and efflux pump. However, no other IVM resistance-associated genes were characterized by conventional methods or strategies. In the present study we adopted a new strategy, i.e. using genome-wide single nucleotide polymorphism (SNP) analysis based on 2b-RAD sequencing, for discovering SNPs markers across the genomes in both IVM susceptible and resistant isolates of *H. contortus* and identifying potential IVM resistance-associated genes.

**Results:**

We discovered 2962 and 2667 SNPs within both susceptible and resistant strains of *H. contortus*, respectively. A relative lower and similar genetic variations were observed within both resistant and susceptible strains (average *π* values were equal to 0.1883 and 0.1953, respectively); whereas a high genetic variation was found across both strains (average *π* value was equal to 0.3899). A significant differentiation across 2b-RAD tags nucleotide sites was also observed between the two strains (average F_ST_ value was equal to 0.3076); the larger differences in average F_ST_ were observed at SNPs loci between coding and noncoding (including intronic) regions. Comparison between resistant and susceptible strains revealed that 208 SNPs loci exhibited significantly elevated F_ST_ values, 24 SNPs of those loci were located in the CDS regions of the nine genes and were likely to have signature of IVM directional selection. Seven of the nine candidate genes were predicted to code for some functional proteins such as potential IVM target and/or efflux pump proteins, component proteins of receptor complex in membrane on neuromuscular cells, and transcriptional regulation proteins. Those genes might be involved in resistance to IVM.

**Conclusions:**

Our data suggest that candidate genes putatively associated with resistance to IVM in *H. contortus* may be identified by genome-wide SNP analysis using 2b-RAD sequencing.

**Electronic supplementary material:**

The online version of this article (doi:10.1186/s13071-016-1959-6) contains supplementary material, which is available to authorized users.

## Background

Parasite nematodes are major causes of morbidity in sheep and cattle. The infections can decrease production of meat and milk in those livestock animals. No vaccines are available for these diseases by far and major control measures rely on the use of anthelmintic drugs. The excessive and uncontrolled use of anthelmintics for the treatment of nematode diseases has led to widespread resistance in livestock nematodes [1]. Clearly, there is an urgent need for better management of drug-use in nematode control. To this end, development of molecular markers for anthelmintic resistance diagnosis is an attractive option for improvements in drug-use decisions [2]. In addition, identification of resistance markers can also help increase our understanding of mechanisms of drug effects, and provide the basis for development of novel anthemintics [2, 3].

Ivermectin (IVM) belongs to the macrocyclic lactone (ML) family of antiparasiticides. Introduced into the market in the early 1980s IVM has been widely used due to its broad-spectrum activity for the control of parasitic nematodes and ectoparasites in animals. As a consequence, IVM resistance has become widespread in nematodes of livestock [3]. Unfortunately, our understanding of the molecular mechanisms underlying resistance to IVM remains far from complete, despite some early indications that resistance to IVM may be due to specific polymorphisms in the drug target receptors, e.g. glutamate-gated chloride ion channel receptors (GluCIRs) [4, 5]. To date, there are no yet mutations identified that can explain the observed resistance to IVM in most field isolates of the parasitic nematode species [2]. In more recent years, studies on the likely genetic mechanisms of resistance suggest that ivermectin resistance in nematodes is multi-genic in nature [6, 7]. Therefore, it is important to discover novel genes associated with resistance to ivermectin for evaluating and testing most cases of field resistance in nematode species. One of the most important research priorities for anthelmintic resistance is to identify mutations in parasite genes that give rise to modification of drug target or nontarget-dependent development of resistance. These studies often examine a small number of loci, sometimes only one, and result in the missed detection for resistance-associated genes, and candidate gene studies are also based on prior assumptions about possible mechanisms of resistance. This situation has major limitations in identifying novel resistant genes and unsuspected mechanisms of resistance [2].

Recent progress resulting in genomic data available for some parasitic nematode species [8, 9], together with the advances of next generation sequencing (NGS) methods for genome-wide genetic marker discovery and genotyping, make it possible for researchers to screen out potential drug resistance genes in the whole genome scale [2, 10]. The principle of candidate gene discovery mentioned above is based on the genome-wide association study (GWAS), also known as whole genome association study (WGAS), which looks for associations between DNA sequence variants and phenotypes of interest. Conventional approach is to type thousands to hundreds of thousands of single nucleotide polymorphisms (SNPs), known as genetic markers, across the genome of interest, and compare the differences of these genetic markers between one case group and a control group, and identify regions/loci of genome/genes with variant genetic markers which are likely to be associated with traits like diseases or drug resistance [11].

2b-RADseq is a restriction site-associated DNA (RAD) sequencing based on sequencing the uniform fragments produced by type IIB restriction end nucleases, it produces high coverage of homologous SNP loci of fixed length, and provides a powerful method for genome-wide SNPs discovery and genomic studies at the population level [12, 13]. 2b-RAD is also well suited to identify genomic regions/loci under selection because of the uniform high density of markers across genomes [14]. It is a cost-effective method, and can be used in routine experimental laboratory. The aim of this study was to provide a preliminary “proof of principle” that candidate anthelmintic resistance-associated genes could be identified by identifying genome-wide signatures of drug selection. To this end, we first applied the 2b-RAD technique to discover thousands of SNPs in both susceptible and resistant strains of *Haemonchus contortus* to ivermectin, and investigated the patterns of genetic diversity and population differentiation across genome of the two strains using above SNPs markers; then we examined the variation in SNP allele frequencies which can be quantified by the statistic F_ST_ between the two strains, and identified candidate resistance-associated genes with signatures of anthelmintic selection by analyzing SNPs loci that exhibited significantly elevated F_ST_ values between the resistant and susceptible strains.

## Methods

### Collection of *Haemonchus contortus*

The susceptible strain of *H. contortus* was originally obtained from Australian and maintained for 2 years in sheep in Huazhong Agricultural University; a resistant strain of *H. contortus* to ivermectin was originally obtained from Moredern Institute of England and maintained for 6 years in sheep in Inner Mongolia Agricultural University. We first tested whether the above-mentioned strains were susceptible or resistant to IVM by using Larval Development Assay (LDA) [15–17]. Indigenous male goats at the age of 3 months were transported from pasture to pens and treated with ivermectin (at dosage of 0.4 mg/kg) and albendazole (at dosage of 30 mg/kg). Each goat was housed in a single pen, and had a free access to a commercial-concentrated-feeding-stuff and water. After 7 days, faecal samples were collected and examined by a modified McMaster technique. Twenty days later nematode egg counts of all animals were found to have negative values (mean faecal egg count of 0 eggs per gram) [18]. One goat was infected with approximately 7000 of the third-stage larvae of susceptible or resistant isolates of *H. contortus*, respectively. On day 30 post-infection, egg counts were examined, eggs were collected, and 100 eggs were used for LDA in each of both experimental and control groups. The results showed that the susceptible strain was very sensitive to ivermectin; whereas resistant strain had a lethal dose of 428.38 ng/ml (LD99) (see Additional file 1: Tables S1 and S2). The results of aforementioned assays indicated that the two strains could be used as the resources of worm samples in the following experiments. On day 15 after examination of LDA, the animals were slaughtered and adult worms were collected for further use.

### Library construction and sequencing

2b-RAD libraries were prepared for *H. contortus* samples by following the protocol developed by Wang et al. [19]. Briefly, each of six genomic DNA samples (3 samples from each of the above-mentioned two strains of *H. contortus*) was extracted by phenol-chloroform method using GenElute Genomic DNA Miniprep Kit (Sigma-Aldrich, Shanghai, China); each sample contained a pool of four adult individuals. DNA from each sample was digested using BsaXI, then verified and separated on agarose gel. Next, library-specific adaptors and the digestion products were linked with T4 DNA ligase. Ligation products were amplified by PCR and the target band was excised from a 2% agarose gel. Finally, sample-specific barcodes were introduced by PCR with platform-specific barcode-bearing primers. PCR products were purified using QIA quick PCR purification kit and then pooled for sequencing using the Illumina HiseqXTen platform. All of the 2b-RAD sequences were archived in the NCBI SRA database (Assay ID: ss2137098375–2137102521).

### Sequence data processing, SNPs discovery and identification of candidate resistance-associated genes

We used Stacks tool with default parameters described by Catchen et al. [20] to conduct the data processing the raw 2b-RAD sequences, discovery of SNPs, patterns of genetic diversity and population differentiation across genome of the two strains and identification of candidate resistance-associated genes. Briefly, billions of raw reads were filtered and cleaned, the data cleaned were aligned to a reference genome of *H. contortus* (ftp://ftp.sanger.ac.uk/pub/pathogens/Haemonchus/contortus) which was a version of draft assembly consisting of 67,687 contigs linked into 26,044 scaffolds of total length 370 Mb, and SNPs markers were identified across all of scaffolds of both strains, and density of SNPs was calculated across nucleotide sites for which sequence information was generated. We calculated population genomic statistics according to method described by Hohenlohe et al. [21] using Stacks tool, we measured the degree of polymorphism within populations with statistic nucleotide diversity *π* (equivalent to expected heterozygosity) and differentiation among populations with statistic fixation index F_ST_. We calculated average values of F_ST_ across whole-genome using a kernel smoothing approach described by Hohenlohe et al. [21], and the feature of SNPs distribution was examined by comparison of average F_ST_ values of SNPs that were located in three different regions (CDS, intronic and noncoding regions) according to the approach described by Akey et al. [22]. We identified SNPs loci with signature of selection by selecting SNPs sites that exhibited significantly elevated F_ST_ values between the resistant and susceptible strains (Smoothed AMOVA F_ST_ > 0.4652 and *P* < 0.16). We identified candidate resistance-associated genes by using following criteria: (i) the genes contained above-mentioned SNPs loci with signature of selection; (ii) those SNPs were located in CDS region and were found in only resistant strain; and (iii) the genes had been annotated. Functional information and annotation of the genes were analyzed by interrogating WormBase.

## Results

### SNPs discovery and their features of distribution

To efficiently discover SNPs in both susceptible and resistant strains of *H. contortus* to ivermectin, we adopted 2b-RAD technique. After raw reads data were filtered and processed by using Stacks software, 2962 and 2667 SNPs were identified within both susceptible and resistant strains of *H. contortus*, respectively (Additional file 2: Table S3). To further determine whether above-mentioned SNPs are representatives of SNPs across the genome, we analyzed their features of distribution. As described by Laing et al. [8], draft assembly of *H. contortus* genome consisting of 67,687 contigs linked into 26,044 scaffolds with a total length of 370 Mb, we observed that a total number of 2,176,234 nucleotide sites for which sequence information was generated, could be aligned to 9258 scaffolds of total length 348.936639 Mb (Additional file 2: Table S3), hence the total number of nucleotide sites accounted for about 0.6% of the *H. contortus* genome (Table 1). In addition, almost half of the nucleotide sites were aligned to the top 1000 scaffolds which had almost half of length of the genome. It was the case for the distribution of SNPs identified across the nucleotide sites (Table 1). We also calculated the density of these SNPs. Since 4873 SNPs identified were distributed across these 2,176,234 nucleotide sites, therefore the density of SNPs across the nucleotide sites was about 1/447 bp (Table 1). This density is lower than that of SNPs across the genome described by Gilleard et al. [23]; these authors provided some data on the level of SNPs across the whole genome in several laboratory strains with the density of the SNPs of 1/202–1/283 bp. The low density of SNPs identified in this study may be due partly to the fact that number of worm samples sequenced is small. Overall, these findings suggest that the distribution of SNPs across the nucleotide sites is similar to that of SNPs across the genome.

**Table 1:** Nucleotide sites and SNPs identified in scaffolds

Scaffolds	Length^a^	Sites^b^	IR^c^	LS	IR_LS	Density of SNPs^d^	Site content^e^
1 to 1000	171,808,466	1,047,366	1381	1465	2454	1/427	0.61%
Others	177,128,173	1,128,868	1286	1497	2419	1/467	0.64%
Total (9258)	348,936,639	2,176,234	2667	2962	4873	1/447	0.62%

^a^Total length (bp) of genome

^b^The total number of nucleotide sites for which sequence information was generated in at least one sample, after trimming restriction enzyme recognition sequence

^c^The remaining columns give the number of single-nucleotide polymorphisms identified within each population. IR population is resistant strain of *H. contortus*, LS is susceptible strain of *H. contortus* and IR_LS is 2 populations combined

^d^Density of SNPs = No. of IR_LS/No. of sites

^e^Site content = No. of sites/Total length (bp) of genome

Furthermore, we analyzed the feature of distribution of the SNPs identified indifferent functional regions (i.e. CDS, intronic and noncoding), and found that the larger differences in average F_ST_ were observed between coding and noncoding as well as intronic SNPs (Table 2), which is consistent with the finding described by Akey et al. [22] who observed a similar feature of distribution of F_ST_ in human genes. Taken together, the SNPs identified in this study can provide an excellent fractional representation of the total of SNPs across the entire genome of *H. contortus* and can be used in further analysis.

**Table 2:** Average F_ST_ as a function of SNP category

	*N*	Average F_ST_	SE
CDS	484	0.2700104	0.011941471
Intro	847	0.3163460	0.009877181
Non-coding	1472	0.3069602	0.007520325

*Abbreviations*: *N* the number of SNPs within each region; *SE* standard error

### Genome-wide estimates of genetic diversity and population differentiation

**Fig. 1:**
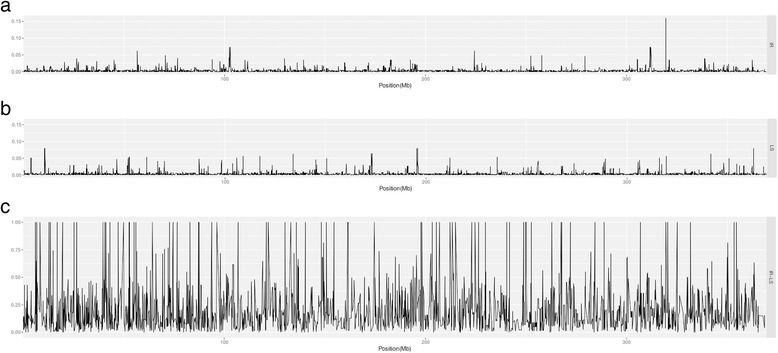
Patterns of genetic diversity and population differentiation distributed across the genome. **a** Genome-wide patterns of nucleotide diversity for resistant strain of *H. contortus*. **b** Genome-wide patterns of nucleotide diversity for susceptible strain of *H. contortus*. **c** Genome-wide differentiation among populations of susceptible and resistant strains of *H. contortus*

To understand whether the susceptible and resistant strains have been subject to different selection pressures, we analyzed genetic diversity and population differentiation within or among the two strains using these SNPs across genome (Fig. 1). We identified lower and similar genetic variation within strains, the average genetic diversity *π* values were equal to 0.1883 and 0.1953 within the susceptible and resistant strains, respectively; and a significant genetic variation was observed across both strains, the average *π* value was 0.3899 (Table 3). This finding is in agreement with previous studies of genetic variation within and among susceptible and resistant populations in the field [24]. A significant differentiation across 2b-RAD tags nucleotide sites was also observed between the two strains (Fig. 1c), the average F_ST_ value was equal to 0.3076 (Table 3). These estimates indicated that the susceptible and resistant strains had been affected by different selection and provided a basis for further identification of a genome-wide signature of selection.

**Table 3:** Pairwise nucleotide diversity and population differentiation among two *H. contortus* populations. Above the diagonal: the average nucleotide diversity (π) in each combined pair of the populations; along the diagonal: π within each single population; below the diagonal: average F_ST_ between the two populations

	IR	LS
IR	0.1883	0.3899043
LS	0.3076215	0.1953

*Abbreviations*: *IR* resistant *H. contortus*; *LS* susceptible *H. contortus*

### Identification of candidate resistance-associated genes

To identify candidate genes that could be subject to IVM selection, we first selected those SNPs loci whose F_ST_ values were significantly greater than that of the genome-wide average 0.3076 (*F*_ST_ > 0.4652 and *P* ≤ 0.16) (see Methods), and found that a total of 208 SNPs loci met this criterion (Additional file 3: Table S4), of which 24 loci were located in the CDS regions and the genes contained these loci had annotation information (Table 4). Then, among these 24 SNPs loci we selected those SNPs that were resistant strain-specific (i.e. only presented in resistant strain) for further analysis, we obtained nine SNPs loci that may have been affected by IVM selection (Table 4). In addition, we further analyzed the functional information of the genes that contained these nine SNPs loci by interrogating wormbase and relevant literatures, finally we identified seven candidate resistance-associated genes that encompassed eight SNPs loci (Table 5).

**Table 4:** SNPs loci that exhibit significantly elevated F_ST_ value, identified in CDS regions

Chr	BP^a^	Smoothed AMOVA F_ST_	Smoothed AMOVA F_ST _ *P*-value	Gene ID
Scaffold_11180	1238	1	< 0.05	cds:HCOI00022400.t1
Scaffold_1311	4966	1	< 0.05	cds:HCOI00277600.t1
Scaffold_1311	4972	1	< 0.05	cds:HCOI00277600.t1
Scaffold_1409	28,964	1	< 0.05	cds:HCOI00335100.t1
Scaffold_148	58,650	0.750886831	< 0.05	^b^cds:HCOI00506600.t3
Scaffold_148	65,574	0.755702686	< 0.05	^b^cds:HCOI00506600.t3
Scaffold_1483	47,819	1	< 0.05	^b^cds:HCOI00378500.t1
Scaffold_1511	40,561	1	< 0.05	cds:HCOI00392800.t1
Scaffold_1595	23,974	1	< 0.05	cds:HCOI00449900.t1
Scaffold_207	42,678	1	< 0.05	cds:HCOI00808400.t1
Scaffold_2990	2020	1	< 0.05	^b^cds:HCOI01034200.t1
Scaffold_340	169,069	1	< 0.05	^b^cds:HCOI01315600.t1
Scaffold_340	169,075	1	< 0.05	cds:HCOI01315600.t1
Scaffold_3511	5425	1	< 0.05	^b^cds:HCOI01200700.t1
Scaffold_3558	3252	1	< 0.05	cds:HCOI01210600.t1
Scaffold_3989	17,378	1	< 0.05	cds:HCOI01318200.t3
Scaffold_4175	4946	1	< 0.05	^b^cds:HCOI01355700.t1
Scaffold_4362	13,965	1	< 0.05	^b^cds:HCOI00703000.t1
Scaffold_4362	13,975	1	< 0.05	cds:HCOI00703000.t1
Scaffold_4362	13,977	1	< 0.05	cds:HCOI00703000.t1
Scaffold_645	29,323	0.784867758	< 0.05	cds:HCOI01894100.t1
Scaffold_6841	192	1	< 0.05	cds:HCOI01753600.t1
Scaffold_774	42,481	1	< 0.05	^b^cds:HCOI02035600.t2
Scaffold_793	2897	0.465277778	0.16	^b^cds:HCOI02054500.t1

^a^Base position of SNP loci located in a given scaffold

^b^The SNPs that are resistant strain-specific and only present in a resistant strain

**Table 5:** Candidate genes related to IVM resistance, identified within CDS regions that exhibit significantly elevated F_ST_ value

Scaffold	BP^a^	Gene ID	Annotation
Scaffold_148	58,650	cds:HCOI00506600.t3	Low density lipoprotein-receptor domain containing protein
Scaffold_148	65,574	cds:HCOI00506600.t3	Low density lipoprotein-receptor domain containing protein
Scaffold_1483	47,819	cds:HCOI00378500.t1	4Fe-4S ferredoxin and ABC transporter domain containing protein
Scaffold_340	169,069	cds:HCOI01315600.t1	RNA polymerase-associated protein RTF1
Scaffold_3511	5425	cds:HCOI01200700.t1	L27-1 and PDZ and Src homology-3 and guanylate kinase domain containing protein
Scaffold_4362	13,965	cds:HCOI00703000.t1	AIR synthase related protein domain containing protein
Scaffold_774	42,481	cds:HCOI02035600.t2	WW Rsp5 WWP and FF domain containing protein
Scaffold_793	2897	cds:HCOI02054500.t1	Neurotransmitter-gatedion-channel ligand-binding

^a^Base position of SNP loci located in a given scaffold

After analyzing functional information of relevant genes according to the Wormbase data and references, we selected those genes that might take part in changes in the drug receptor or modulation of drug concentration as candidate genes; they may code for some functional molecules such as potential IVM target and/or efflux pump proteins, component proteins of receptor complex in membrane on neuromuscular cells, and transcriptional regulation proteins. We found that the four molecules encoded by HCOI02054500, HCOI00378500, HCOI01200700 and HCOI02035600 genes, respectively, were likely to be a potential IVM target or efflux pump proteins. According to Wormbase (http://parasite.wormbase.org/Haemonchus_contortus_prjeb506/Info/Index) we found that the protein encoded by the HCOI02054500 gene was likely to have the function similar to that of GABA receptors; the protein encoded by the HCOI00378500 gene may participate transfer of energy and substrates; and the two proteins, encoded by the HCOI01200700 and HCOI02035600 genes may participate in the formation of receptor complex in membrane on the neurons and or muscle cells of *H. contortus*, respectively. We also found that the protein encoded by the HCOI00506600 gene may take part in lipid metabolism and influence the nematode *P*-*gp* activity. Meanwhile, we inferred that enzyme encoded by HCOI00703000 was likely to be a new class of ATP-binding proteins and may be related to the drug metabolism and detoxification. A recent study had indicated that some genes responsible for transcriptional regulation may also be involved in IVM resistance [25]; according to the annotation, we inferred that molecule encoded by the HCOI01315600 gene was likely to play a role in the regulation of transcription of ABC transporter genes in *H. contortus*.

As for the HCOI01034200 and HCOI01355700 genes which encode glycoside hydrolase domain containing protein and peptidase S9 domain containing protein, respectively, we were unable to provide evidence that the proteins encoded by the two genes are likely to be responsible for the resistance to IVM in *H. contortus*. Hence, we did not categorize them into our list of candidate IVM resistance-associate genes.

## Discussion

*Haemonchus contortus* has shown a great ability to develop resistance to all the anthelmintic drug classes including macro cyclic (ML) lactones, e.g. IVM [3]. Previous studies have shown that changes in target site and drug efflux pathways, e.g. over expression of a number of P-glycoprotein genes may play roles in resistant isolates to IVM, but no definitive mechanism could explain the observed field resistance [2]. Recent investigations on the likely genetic mechanisms of resistance have indicated that multigenic basis and another mechanism may have contributed to ML resistance in *H. contortus* [7, 26, 27]. Therefore, discovery and identification of novel molecules responsible for ML resistance has caught more attention. Conventional strategy for discovery of candidate anthelmintic resistance-associated molecules in *H. contortus* is to draw on the experience of similar work in mammals and *C. elegans* worms [28–31]. In this present study, we adopted a new strategy for the first time, i.e. genome-wide scan based on SNPs analysis to identify other candidate genes or molecular markers associated with resistance to IVM in *H. contortus*.

Our strategy is based on a basic principle of population genomics, i.e. under the condition of selective neutrality, F_ST_ statistic of population is determined by genetic drift, which will affect all loci across the genome in a similar fashion, but naturals election (e.g. under pressure of drug) is a locus-specific force that can cause systematic deviations in F_ST_ values for a selected gene and nearby genetic markers [32–34]. Therefore, for F_ST_ statistic we can calculate both a genome-wide average and outliers by population genomics methods, the genome-wide average provides a baseline value of neutral processes, and outliers from the background are likely to be the locus-specific signature of positive directional selection [22, 32]. It is noteworthy to mention that such strategy has been reported in several recent studies, e.g. Cheeseman et al. [23, 35] reported that they were able to identify genome regions underlying *Artemisia* in resistance in malaria by mapping genome-wide divergence (F_ST_) between drug-resistant and drug-sensitive parasites . Tennessen et al. [36] also adopted a similar strategy to identify a region of the snail *Biomphalaria glabrata* genome that correlates with resistance to *Schistosoma mansoni* infection. So it is a feasible that this approach can be applied to our study.

In order to obtain genome-wide DNA markers, we first identified thousands of SNPs from 2b-RAD tag sequences for both resistant and sensitive to IVM worms. Relative lower density (about 7.56 SNPs per Mb) and genetic diversity of the SNPs were observed across the whole genome within both strains, this is due partly to the fact that the number of worm samples (similar to the term census population size) sequenced is small, this is consistent with the results of previous investigations indicating that the high levels of genetic diversity within *H. contortus* populations are largely due to their large census population sizes [24]. On the other hand, the factors that adult worms from each of the two strains were collected from a single host (known as infra population) may influence genetic diversity. We also observed a similar result within two field infra populations in Sichuan and Inner Mongolia China (unpublished data). In spite of the limitation mentioned above, some features of the population genetic structure of *H. contortus* can still be demonstrated by statistical analysis of these SNPs, e.g. in the present study, a similar level of genetic diversity was observed within both resistant and susceptible strains, consistent with previous studies showing that *H. contortus* field populations that are resistant to anthelmintic drugs seem to have a similar level of overall genetic diversity as susceptible populations [24]. In addition, Akey et al. [22] interrogated a high-density SNP map to analyze signatures of natural selection in the human genome, and found that the largest difference in average F_ST_ could be observed between coding and noncoding SNPs due to natural purifying selection (F_ST_ for coding region < F_ST_ for noncoding region). In spite of the low-density of SNPs obtained in the present study, we were also able to obtain a similar result. These results suggest that those SNPs from 2b-RAD tag sequences can provide an excellent fractional representation of the total of SNPs in the entire genome of *H. contortus*, and can be used to reveal the locus-specific signature of drug selection.

In fact, we found that 208 SNP loci exhibited significantly elevated F_ST_ value, of which 24 loci were located in CDS region, the others within intron and another noncoding region, those genes with significant variation in SNP allele frequencies should be considered as potential IVM resistance-associated genes. Among those genes, although our study failed to find known candidate IVM resistant genes such as *Hco*-*glc* (encoding IVM target glutamate-gated chloride channel receptor, GluClR) and *Hco*-*pgp* (encoding ABC transporter membrane protein P-glycoprotein for IVM efflux pumps) [6, 8, 37, 38], we were still able to identify some genes with functions of drug target and efflux pump similar to that of *Hco*-*glc* and *Hco*-*pgp*, for example, HCOI02054500 and HCOI00378500 genes. According to the annotation by Wormbase parasite (http://parasite.wormbase.org/Haemonchus_contortus_prjeb506/Info/Index), HCOI02054500 gene was predicted to encode an uncharacterized protein with a molecular function of extracellular ligand-gated ion channel activity, and to have a *C. elegans* or thologue lgc-36 which is an or tholog of members of the human GABR [Gamma-amino butyric acid (GABA) A receptors] family including GABRR1, suggesting that HCOI02054500 is likely to have the function similar to that of GABA receptors. Meanwhile, the HCOI00378500 gene was annotated as encoding 4Fe-4S ferredoxin and ABC transporter domain containing protein; this protein belongs to ATP-binding cassette sub-family E member 1 (ABCE1); it was predicted to have ATP binding activity and ATPase activity, and might be involved in nematode larval development and reproduction, suggesting that this protein is likely to mediate transfer of energy and substrates. As we have known, IVM is believed to act by opening glutamate-gated chloride channels and GABA-gated channels in nematodes neurons or muscle cells which leads to a permanent hyperpolarisation and an inhibitory paralysis of the cells. Early reports on the mechanism of IVM also indicated that changes in GluCl and GABA receptors may be implicated in ML resistance in laboratory-selected resistant *H. contortus* [2]. In addition, multidrug resistance ABC transporters are essential for many cellular processes that require the transport of substrates across cell membranes; and the over expression of those transporter genes can be rapidly and transiently induced following IVM and moxidectin treatment in *H. contortus* [39], which leads to therapy failure by decreasing drug concentration at the target. Some early molecular analyses also demonstrated that polymorphisms in *H. contortus P*-*gp* genes may have been associated with resistance to MLs [2]. All these data suggest that genetic changes in drug sites and drug efflux pump transporters of *H. contortus* may have been implicated in IVM resistance. Therefore, we speculate that the two novel genes identified in this study may have contributed to the IVM resistance in *H. contortus*, and can be selected as candidate IVM resistance-associated genes.

Among the candidate genes screened in this study, it is worth to note that the HCOI00703000 gene (http://parasite.wormbase.org/Haemonchus_contortus_prjeb506/Info/Index) is annotated to code for AIR synthase-related protein domain containing protein which probably contains ATP-binding site; this protein is believed to represent a new class of ATP-binding proteins. This kind of protein (enzyme) was reported to catalyze the conversion of formylglycinamide ribonucleotide (FGAM) and ATP to AIR, ADP and Pi, the fifth step in *de novo* purine biosynthesis. Moreover, previous studies have suggested that a pyrrolo [2,3-d] pyrimidine folate analog inhibits variety of human folate-requiring enzymes, including PurN and PurH, and purine biosynthesis pathway was thought as a chemotherapeutic target [40]. Whether the protein encoded by the HCOI00703000 gene in *H. contortus* is related to the drug metabolism and detoxification or even can be one of the drug targets, remain to be further validated.

It is also interesting to noting that the HCOI00506600 gene (http://parasite.wormbase.org/Haemonchus_contortus_prjeb506/Info/Index), coding for low density lipoprotein-receptor domain containing protein, has significantly shown higher genetic diversity, and may participate in the regulation of lipid metabolism which could lead to the drug resistance *via* multi-drug resistance (MDR) proteins (e.g. *P*-*gp*) [41]. In facts, the *P*-*gps* have been localized to the biological membranes and the membrane environment has been shown to modulate their activity. The membrane environment is mainly composed of lipids. Low density lipoprotein-receptor can mediate the endocytosis of cholesterol-rich LDL, and change membrane environment [42]. According to the studies by Riou et al. [43], changes in the cholesterol content in *H. contortus* eggs induced changes in benzimidazoles and ivermectin anthelmintic resistance; cholesterol depletion gave increased resistance and cholesterol loading gave decreased resistance; and the effect is likely to be correlated with changes in the function of membrane P-glycoprotein [44]. Above experimental evidence indirectly suggests that polymorphism of the HCOI00506600 gene may influence the nematode *P*-*gp* activity *via* modulating lipid composition of membrane and this gene may be involved in IVM resistance in *H. contortus*. Hence, this gene should be selected as a potential IVM resistance-associated gene.

We also found that the HCOI01200700 gene, encoding L27-1 and PDZ and Srchomology-3 and guanylate kinase domain containing protein, was shown to have significant change of SNP sites. According to the annotation (http://parasite.wormbase.org/Haemonchus_contortus_prjeb506/Info/Index), this gene has a *C. elegans* orthologue *dlg*-*1* that encodes a membrane-associated guanylate kinases (MAGUK) protein [45]. They are a super family of proteins, have emerged as a key element in the organization of protein complexes in specialized membrane regions. These proteins are characterized by the presence of multiple protein-protein interaction domains including PDZ and SH3 domains. They are located either on the pre- and/or post-synaptic sides of synapses or at cell-cell adhesion sites of epithelial cells. MAGUK proteins can interact with glutamate receptors and various ionic channels, they have ability to form protein-protein interactions with cytoskeleton proteins, microtubule/actin-based machinery and molecules involved in signal transduction [46]. Meanwhile, we found that the HCOI02035600 gene probably has similar function to HCOI01200700 gene (http://parasite.wormbase.org/Haemonchus_contortus_prjeb506/Info/Index), this gene, coding for WW- Rsp5- WWP- and FF domain-containing protein, was predicted to be required for embryonic viability and for normally high rates of postembryonic growth. The WW domain has been originally discovered as a short conserved region in a number of unrelated proteins, e.g. dystrophin, amultidomain cytoskeletal protein that is thought to have multiple functions including involvement in membrane stability, transduction of contractile forces to the extracellular environment and organization of membrane specialization. Mutations in the dystrophin gene lead to muscular dystrophy of Duchenne or Becker type [47]. Therefore, we inferred that proteins encoded by HCOI01200700 and HCOI02035600 genes may participate in the formation of receptor complex in membrane on the neurons and/or muscle cells of *H. contortus*, and these receptors may be anthelmintic targets or efflux pumps, suggesting that both HCOI01200700 and HCOI02035600 genes may indirectly be implicated in resistance to IVM.

A recent study has indicated that some ABC transporter genes were shown to have significant increase in transcription following 3 h exposure to both IVM and LEV in the resistant *H. contortus* isolate, suggesting that some genes responsible for transcriptional regulation may also be involved in IVM resistance [25]. But up to now, no transcriptional regulation genes that may be related to IVM resistance were identified. In the present study we found that the HCOI01315600 gene (http://parasite.wormbase.org/Haemonchus_contortus_prjeb506/Info/Index) was likely to be one of this kind of genes. This gene was annotated as coding for RNA polymerase-associated protein RTF1 and likely to play a role in regulation of transcription. Tenney et al. [48] showed that *Drosophila* Rtf1 (dRtf1) protein was required for proper gene expression and development, and also participated in histone methylation and Notch signaling; these transcriptional regulation functions of Rtf1 *via* the Paf1 complex are highly conserved among eukaryotes [48]. We inferred that the functions of *H. contortus*Rtf1 protein was likely to be similar to that of dRtf1and may be indirectly implicated in resistance to IVM by increasing the transcription levels of ABC transporter genes in resistant strains, a prediction that remains to be further confirmed experimentally.

## Conclusions

In conclusion, our data suggest that candidate genes putatively associated with resistance to IVM in *H. contortus* may be identified by genome-wide SNP analysis using 2b-RAD sequencing. Seven candidate genes were predicted to code for some functional molecules such as potential IVM target and/or efflux pump proteins, component proteins of receptor complex in membrane on neuromuscular cells, and transcriptional regulation proteins; and might be involved in resistance to IVM *via* the mechanisms of changes in the drug receptor or modulation of drug concentration in *H. contortus*. These findings provide not only an indirect evidence for multigenic model of resistance but also a theoretical basis for further experimental validation of these novel IVM resistance-associated proteins.

## Additional files

Additional file 1: Table S1. Larval development assay to determine ivermectin resistance in the susceptible strain of *H. contortus*. **Table S2**. Larval development assay to determine ivermectin resistance in the resistant strain of *H. contortus*. (DOCX 16 kb)
Additional file 2: Table S3. Nucleotide sites and SNPs identified on each scaffold of both susceptible and resistant strains of *H. contortus*. (XLSX 345 kb)
Additional file 3: Table S4. A complete list of 208 SNPs loci exhibits significantly elevated F_ST_ value. (XLSX 16 kb)

## Abbreviations

ABCE1: ATP-binding cassette sub-family E member 1
CDS: Coding sequence
FGAM: Formylglycinamide ribonucleotide
GABA: Gamma-amino butyric acid
*glc*: Glutamate-gated chloride
GluCIRs: Glutamate-gated chloride ion channel receptors
GWAS: Genome-wide association study
*Hco*: *Haemonchus contortus*
IR: Ivermectin resistant
IVM: Ivermectin
LDA: Larval development assay
LS: Ivermectin susceptible
MAGUK: Membrane-associated guanylate kinases
MDR: Multi-drug resistance
ML: Macrocyclic lactone
NGS: Next generation sequencing
*P*-*gp*: P-glycoprotein
RAD: Restriction site-associated DNA
SNP: Single nucleotide polymorphism
WGAS: Whole genome association study
