# Supplementary material for: Genome-wide SNP analysis using 2b-RAD sequencing identifies the candidate genes putatively associated with resistance to ivermectin in Haemonchus contortus
Source: Parasit Vectors. 2017 Jan 17;10:31. doi: 10.1186/s13071-016-1959-6 (PMC5240194; doi:10.1186/s13071-016-1959-6)
Supplement: Additional file 1: Table S1. — Larval development assay to determine ivermectin resistance in the susceptible strain of H. contortus. Table S2. Larval development assay to determine ivermectin resistance in the resistant strain of H. contortus. (DOCX 16 kb) [file 13071_2016_1959_MOESM1_ESM.docx]

**Additional file 1. Table S1** Larval development assay to determine ivermectin resistance in the susceptible strain of *H. contortus*

| Concentration(ng/ml)^1^ | log(concentration) | Inhibition rate of larval growth |
| --- | --- | --- |
| 0 |  | 0.07 |
| 1 | 0.00 | 1.00 |
| 2 | 0.69 | 1.00 |
| 3.9 | 1.36 | 1.00 |
| 7.8 | 2.05 | 1.00 |
| 15.6 | 2.75 | 1.00 |
| 31.25 | 3.44 | 1.00 |
| 62.5 | 4.14 | 1.00 |
| 125 | 4.83 | 1.00 |
| 250 | 5.52 | 1.00 |
| 500 | 6.21 | 1.00 |
| 1000 | 6.91 | 1.00 |

1. Concentrations of ivermectin. The results showed that the susceptible *H. contortus* is sensitive to ivermectin.

**Table S2** Larval development assay to determine ivermectin resistance in the resistant strain of *H. contortus*

| Concentration(ng/ml)^1^ | log(concentration) | Inhibition rate of larval growth |
| --- | --- | --- |
| 0 |  | 0.05 |
| 1 | 0.00 | 0.13 |
| 2 | 0.69 | 0.06 |
| 3.9 | 1.36 | 0.08 |
| 7.8 | 2.05 | 0.06 |
| 15.6 | 2.75 | 0.17 |
| 31.25 | 3.44 | 0.26 |
| 62.5 | 4.14 | 1.00 |
| 125 | 4.83 | 1.00 |
| 250 | 5.52 | 1.00 |
| 500 | 6.21 | 1.00 |
| 1000 | 6.91 | 1.00 |

1. Concentrations of ivermectin. The data were analysed by a logistic regression model to determine the LD_50_ and LD_99_. The results showed that the Regression of equation is y = 0.179x-0.094 (x is log(concentration); y is inhibition rate of larval growth)and R²=0.795. So, the LD_99_ is 428.38ng/ml and the resistant *H. contortus* are resistance to ivermectin.
